# Impaired Mitochondrial Dynamics and Nrf2 Signaling Contribute to Compromised Responses to Oxidative Stress in Striatal Cells Expressing Full-Length Mutant Huntingtin

**DOI:** 10.1371/journal.pone.0057932

**Published:** 2013-03-01

**Authors:** Youngnam N. Jin, Yanxun V. Yu, Soner Gundemir, Chulman Jo, Mei Cui, Kim Tieu, Gail V. W. Johnson

**Affiliations:** 1 Departmentsof Pharmacology and Physiology, University of Rochester, Rochester, New York, United States of America; 2 Department of Biology, University of Rochester, Rochester, New York, United States of America; 3 Department of Anesthesiology, University of Rochester, Rochester, New York, United States of America; 4 Department of Neurology, University of Rochester, Rochester, New York, United States of America; Institut Curie, France

## Abstract

Huntington disease (HD) is an inherited neurodegenerative disease resulting from an abnormal expansion of polyglutamine in huntingtin (Htt). Compromised oxidative stress defense systems have emerged as a contributing factor to the pathogenesis of HD. Indeed activation of the Nrf2 pathway, which plays a prominent role in mediating antioxidant responses, has been considered as a therapeutic strategy for the treatment of HD. Given the fact that there is an interrelationship between impairments in mitochondrial dynamics and increased oxidative stress, in this present study we examined the effect of mutant Htt (mHtt) on these two parameters. STHdh^Q111/Q111^ cells, striatal cells expressing mHtt, display more fragmented mitochondria compared to STHdh^Q7/Q7^ cells, striatal cells expressing wild type Htt, concurrent with alterations in the expression levels of Drp1 and Opa1, key regulators of mitochondrial fission and fusion, respectively. Studies of mitochondrial dynamics using cell fusion and mitochondrial targeted photo-switchable Dendra revealed that mitochondrial fusion is significantly decreased in STHdh^Q111/Q111^ cells. Oxidative stress leads to dramatic increases in the number of STHdh^Q111/Q111^ cells containing swollen mitochondria, while STHdh^Q7/Q7^ cells just show increases in the number of fragmented mitochondria. mHtt expression results in reduced activity of Nrf2, and activation of the Nrf2 pathway by the oxidant tBHQ is significantly impaired in STHdh^Q111/Q111^ cells. Nrf2 expression does not differ between the two cell types, but STHdh^Q111/Q111^ cells show reduced expression of Keap1 and p62, key modulators of Nrf2 signaling. In addition, STHdh^Q111/Q111^ cells exhibit increases in autophagy, whereas the basal level of autophagy activation is low in STHdh^Q7/Q7^ cells. These results suggest that mHtt disrupts Nrf2 signaling which contributes to impaired mitochondrial dynamics and may enhance susceptibility to oxidative stress in STHdh^Q111/Q111^ cells.

## Introduction

Huntington disease (HD) is a devastating inherited neurodegenerative disease caused by a CAG trinucleotide repeat expansion in exon 1 of huntingtin (Htt) gene. Although the mechanisms by which mutant huntingtin (mHtt) causes neurotoxicity have been widely studied, the pathological processes have not yet been fully elucidated. mHtt-induced impairment of the cellular responses to oxidative stress has been suggested as a crucial contributing factor in the progression of HD. Indeed, there is clear evidence of increased oxidative stress in HD. Further, defects in mitochondria, which are both a source of oxidative stress and a target, are apparent in HD and HD models [1], [2].

Neurons are highly dependent on mitochondria for not only energy production but also Ca^2+^ buffering, and reactive oxygen species (ROS) regulation. Studies using mouse and cell models for HD have shown mitochondrial impairment and bioenergetic deficits, reminiscent of the pathological characteristics of HD [2]–[4]. In addition, increased oxidative stress is apparent in HD cases [5]–[7]. Mitochondria from STHdh^Q111/Q111^ cells show impaired function and significant increases in ROS production compared to STHdh^Q7/Q7^ cells [8]–[10]. Our previous studies showed that the PPARγ pathway, which regulates the expression of metabolically important genes, is severely compromised in STHdh^Q111/Q111^ cells which coincides with an increased sensitivity to thapsigargin induced loss of mitochondrial membrane potential (ΔΨm), and increased cell death at higher concentrations of thapsigargin. Activation of the PPARγ pathway attenuated thapsigargin-induced ΔΨm loss and cell death in STHdh^Q111/Q111^ cells [8], [11].

Mitochondria are dynamic organelles that are constantly undergoing fission and fusion, which is essential for normal cellular function. Imbalances between mitochondria fission and fusion have been shown to negatively impact the physiology and viability of neuronal cells [12], [13]. Key mediators of mitochondrial fission/fusion include the GTPases Dynamin-Related protein 1 (Drp1), which is essential for fission, and Optic Atrophy Type 1 (Opa1) and the Mitofusins (Mfn1 and Mfn2) which mediate fusion. mHtt has been reported to directly bind Drp1 and increase its activity, suggesting that this may contribute to the apparent mitochondrial fragmentation and dysfunction [14]. Indeed, fragmentation of mitochondria results in increased ROS production in cell models [15], [16]. Mutations of Opa1 result in autosomal dominant optic atrophy [17]. In addition, the levels of Opa1, as well as Mfn1/2 were shown to be decreased in HD cases relative to controls [18]. There is also data to indicate that alterations in mitochondria morphology in HD enhance cellular susceptibility to apoptosis [14], [19].

Nuclear factor erythroid 2-related factor 2 (Nrf2), a major transcription factor for antioxidant and cytoprotective responses, is normally sequestered in the cytosol by Kelch-like ECH-associated protein 1 (Keap1), an adaptor of a ubiquitin ligase complex, and constitutively degraded through the ubiquitin-proteasome system [20]. Upon oxidative stress, Nrf2 dissociates from Keap1, translocates into nucleus, and binds to antioxidant response elements (AREs), which in turn activates genes related to antioxidant responses such as heme oxygenase-1 (HO-1), NAD(P)H quinone oxidoreductase 1 (Nqo1) and nuclear respiratory factor-1 (Nrf1) [21]–[24]. Activation of Nrf2 signaling has been shown to regulate mitochondrial biogenesis in rat brain, mouse cardiomyocytes, and a sepsis mouse model [23], [25], [26]. Nrf2 signaling or expression is altered in various neurodegenerative diseases and animal models [27], [28]. Pharmacological activation of Nrf2 has beneficial effects in an HD mouse model induced by 3-nitropropionic acid (3-NP), and transgenic mouse models including N171-82Q, R6/2, and YAC128 [29]–[31]. However, whether mHtt affects Nrf2 signaling is not known.

In the present study, we analyzed mitochondrial dynamics and how they are affected by oxidative stress in a striatal cell model of HD. The cells used in this study are homozygous for either the wild type or mutant Htt gene. Although HD is an autosomal dominant disease, the effects of mHtt are often studied in cell and animal models that are homozygous for mHtt to facilitate the dissection of the pathogenic mechanisms involved [1]–[4]. Although these knock-in models are imperfect, they are advantageous in that full-length mHtt, rather than truncated versions of the protein, is expressed and thus may be more informative about disease mechanisms in humans. Nonetheless the fact that they express two copies of the mutant allele, rather than just one as in HD, needs to be carefully considered when evaluating the data obtained with these models.

In this study we found that STHdh^Q111/Q111^ cells exhibit alterations in mitochondrial dynamics compared to STHdh^Q7/Q7^ cells. Determination of static mitochondrial morphology along with analysis of dynamic mitochondrial fusion processes revealed that STHdh^Q111/Q111^ cells exhibit an increase in fragmented mitochondria concomitant with a decrease in fusion. Further STHdh^Q111/Q111^ cells show impairment of the mitochondrial response to oxidative stress. Given that Nrf2 is not only a key regulator for antioxidant system but also an emerging target to counteract mitochondrial dysfunction in many disease models [25], [26], [32]–[34], we investigated whether Nrf2 signaling was compromised in this cell model of HD and found that the presence of mHtt results in defects in Nrf2 signaling. Significant impairment in oxidative stress-induced Nrf2 activation was observed in STHdh^Q111/Q111^ cells. Our study provides important insights into mHtt-induced mitochondrial impairment, with activation of Nrf2 signaling being a potential therapeutic strategy.

## Results

### STHdh^Q111/Q111^ Cells Show Altered Mitochondrial Dynamics

**Figure 1:**
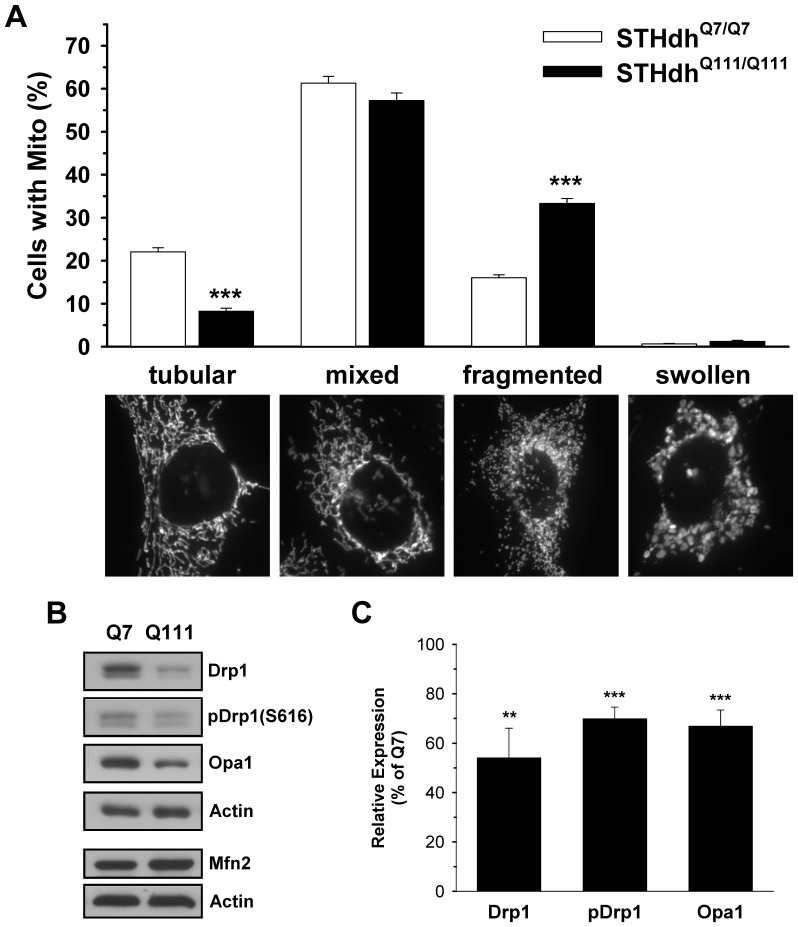
Mitochondrial dynamics is altered in STHdh^Q111/Q111^ cells. *A*, Mitochondria-targeted GFP (mitoGFP), was transfected in the striatal cells to visualize the mitochondria and cells were then assigned to one of four groups based on the morphology of their mitochondria (tubular, mixed, fragmented or swollen). STHdh^Q111/Q111^ cells had more fragmented mitochondria and less tubular mitochondria compared to STHdh^Q7/Q7^ cells, while mixed and swollen mitochondria were at similar levels in the two cell types. More than 200 cells of each cell type were assessed for each independent experiment. n = 3 *B,* Immunoblots revealed that STHdh^Q111/Q111^ cells exhibit reduced levels of Drp1, its active form which is phosphorylated at Ser616, and Opa1 compared to STHdh^Q7/Q7^ cells, whereas Mfn2 appears to be similar in both cell types. *C* **,** Quantitative analysis of immunoblots shows a significant reduction in Drp1, phosphorylated Drp1, and Opa1. n = 4. Data shown are mean ± SE. **P<0.01, ***P<0.001 vs. STHdh^Q7/Q7^.

Mitochondrial morphology is an important component of cellular metabolism and pathogenic processes. Mitochondria are dynamic and display a range of morphologies. Maintaining the appropriate proportion of each mitochondrial morphological state is essential for appropriate functioning of the cell. To gain insight into mitochondrial dynamics in mHtt expressing cells, we transfected mitochondria-targeted GFP into STHdh^Q7/Q7^ and STHdh^Q111/Q111^ cells. Cells were divided into four groups based on the morphology of the mitochondria: tubular, fragmented, mixed, and swollen as previously described [35]. In most cells of both genotypes the mitochondria (about 60%) exhibited mixed forms with predominantly short tubular shapes. Approximately 20% of STHdh^Q7/Q7^ cells had predominantly tubular mitochondria while less than 10% of STHdh^Q111/Q111^ cells showed this mitochondrial morphology. Interestingly, more than 30% of the STHdh^Q111/Q111^ cells presented with fragmented mitochondria which was significantly greater than the STHdh^Q7/Q7^ cells. Both cell types exhibited very few cells with swollen mitochondria (Fig. 1*A*). Similarly, a significant increase in fragmented neuronal mitochondria in the striatum of R6/2 mice was also observed (Figure S1). Next, we examined whether there was a difference in the expression of mitochondrial fusion or fission regulators between the two cell types. Immunoblot analyses revealed that Drp1 levels were significantly lower in STHdh^Q111/Q111^ cells than in STHdh^Q7/Q7^ cells. In addition Drp1 phosphorylated at serine 616, which is a more active form [36], [37], was also lower in STHdh^Q111/Q111^ cells compared to SThdh^Q7/Q7^ cells. Of interest, Opa1 expression was also significantly decreased in STHdh^Q111/Q111^ cells, while Mfn2 appears to be at a similar level between the two cell types (Fig. 1, *B* and *C*). Together these results indicate that proteins involved in the dynamics of mitochondrial fission/fusion are altered in STHdh^Q111/Q111^ cells.

### Mitochondrial Fusion is Impaired in STHdh^Q111/Q111^ Cells

**Figure 2:**
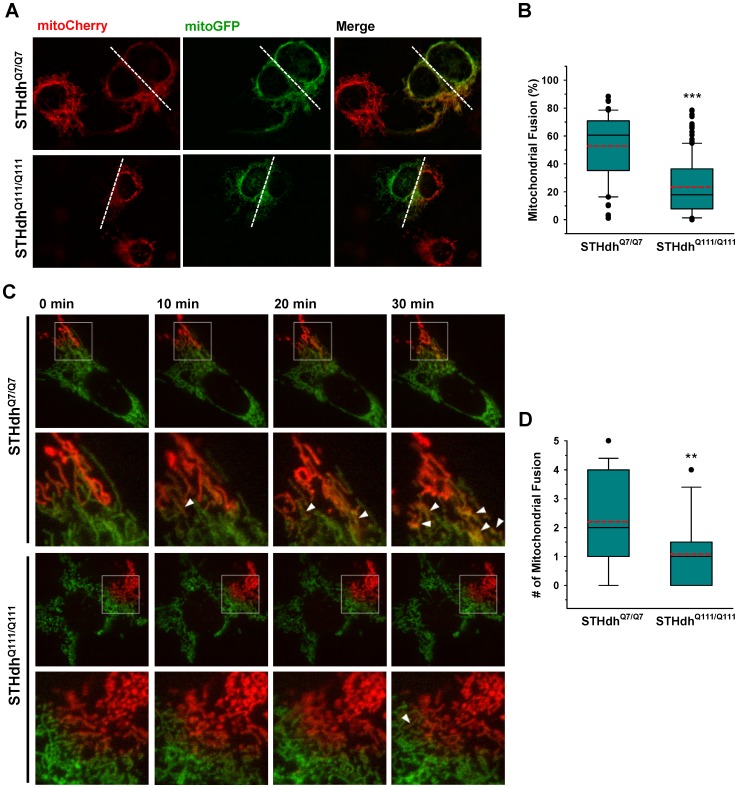
Mitochondrial fusion is significantly reduced in STHdh^Q111/Q111^ cells. *A* **,** Striatal cells expressing mitoCherry or mitoGFP were fused and mitochondrial fusion was analyzed as described in materials and methods. STHdh^Q111/Q111^ cells exhibit substantially lower levels of mitochondrial fusion compared to STHdh^Q7/Q7^ cells. *B* **,** Quantitative analysis reveals that STHdh^Q7/Q7^ cells exhibit ∼60% mitochondrial fusion on average while STHdh^Q111/Q111^ cells display ∼20% fused mitochondria. The data was acquired by analysis of more than 60 cells of each cell type from seven independent experiments. The white dashed line indicates the contact region between the two cells. *C,* To monitor mitochondrial fusion without cell fusion, mitoDendra was expressed in the striatal cells. The mitochondrial fusion process was observed after the photoconversion of green to red fluorescence in a small population of mitochondria as described in methods and materials. The arrow head indicates the yellowish mitochondria formed by the fusion between red and green mitochondria. *D* **,** The quantitative data shows that the mitochondrial fusion rate in STHdh^Q111/Q111^ cells is significantly reduced compared to STHdh^Q7/Q7^ cells. The data was acquired by analysis of more than 20 cells of each cell type from five independent experiments. Data shown are mean ± SE. **P<0.01, ***P<0.001 vs. STHdh^Q7/Q7^.

To gain further insight into dynamic changes in mitochondrial morphology, we investigated whether mitochondrial fusion processes were altered in STHdh^Q111/Q111^ cells compared to STHdh^Q7/Q7^ cells. To this end, striatal cells were fused using PEG after transfection with either mitoCherry or mitoGFP as described in materials and methods. Cell fusion allows two individual cells to share cellular components including mitochondria. If a mitochondrion expressing mitoCherry undergoes fusion with a mitochondrion expressing mitoGFP, the resulting fused mitochondrion becomes yellow. STHdh^Q111/Q111^ cells exhibited significantly lower levels of fused mitochondria compared to STHdh^Q7/Q7^ cells (Fig. 2, *A* and *B*). Since this cell fusion assay requires the use of cycloheximide to prevent de novo protein synthesis which would generate mitochondria containing both mitoCherry and mitoGFP without the fusion process, it can be speculated that inhibition of protein synthesis might differentially affect mitochondrial fusion process in the two cell types. Therefore we used a second method to monitor mitochondrial fusion, a photoconvertible GFP (Dendra) targeted to mitochondria. Dendra, which in the unconverted state fluoresces green, was stably converted in a given area of the cell by light illumination at 400 nm and thus showed red fluorescence. When mitochondria in the cell emitting red fluorescence fuse with mitochondria emitting green fluorescence, the fluorescence becomes yellow. Using this approach, more mitochondrial fusion events were again observed in the STHdh^Q7/Q7^ cells compared to STHdh^Q111/Q111^ cells (Fig. 2, *C* and *D*). Although neither method estimates the mitochondrial fission process, these results provide strong evidence that there is reduction in mitochondrial fusion events in STHdh^Q111/Q111^ cells.

### Oxidative Stress Induces Differential Responses in Mitochondria Morphology

**Figure 3:**
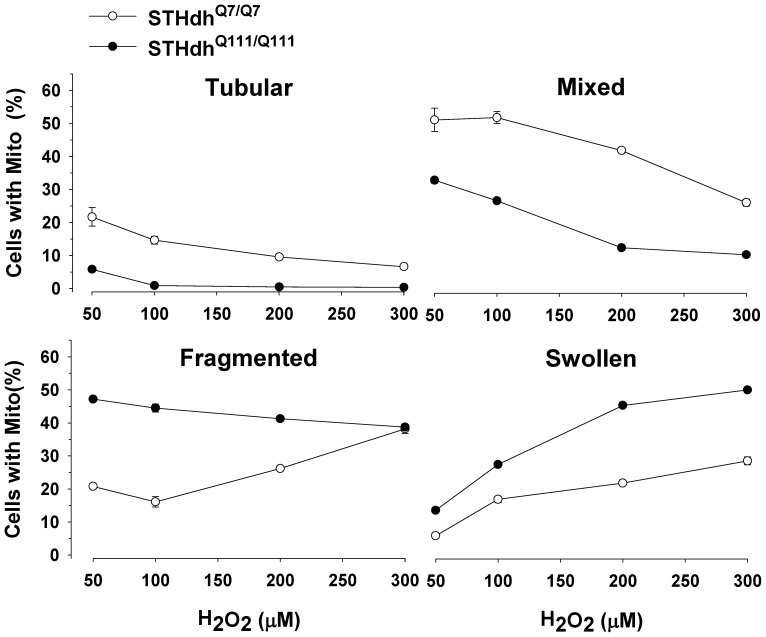
Oxidative stress substantially increases swollen mitochondria in STHdh^Q111/Q111^ cells. mitoGFP was transfected in the striatal cells. H_2_O_2_ treatment results in remarkably different dynamics in mitochondrial morphology between the two cell types. STHdh^Q111/Q111^ cells show faster decline in the tubular and mixed mitochondria compared to STHdh^Q7/Q7^ cells. STHdh^Q7/Q7^ cells exhibit a rapid increase in fragmented mitochondria while STHdh^Q111/Q111^ cells show a slight decrease in fragmented mitochondria. Importantly, STHdh^Q111/Q111^ cells display a dramatic increase in swollen mitochondria whereas STHdh^Q7/Q7^ cells show only a mild increase. n = 4.

It has been shown that mitochondria dynamically change their morphologies in response to oxidative stress [38]–[40]. Therefore, we tested whether the two striatal cell lines showed different pattern changes in mitochondrial morphology in response to H_2_O_2_. In both cell types there were dramatic reductions in tubular and mixed forms of mitochondria in response to H_2_O_2_ in a dose dependent manner (Fig. 3). Importantly, STHdh^Q111/Q111^ cells showed significant increases in swollen mitochondria while the percentage of cells with fragmented mitochondria slightly decreased as the concentration of H_2_O_2_ was increased (Fig. 3). In contrast, STHdh^Q7/Q7^ cells showed a rapid increase in the population of fragmented mitochondria while swollen mitochondria only slightly increased with the increasing concentrations of H_2_O_2_. It is noteworthy that swollen mitochondria are shown to be functionally deficient and indicative of pathogenesis [41], [42]. Therefore, this result suggests that mitochondria in STHdh^Q111/Q111^ cells show greater susceptibility to oxidative stress compared to STHdh^Q7/Q7^ cells.

### Mitochondria in STHdh^Q111/Q111^ Cells are More Oxidized

**Figure 4:**
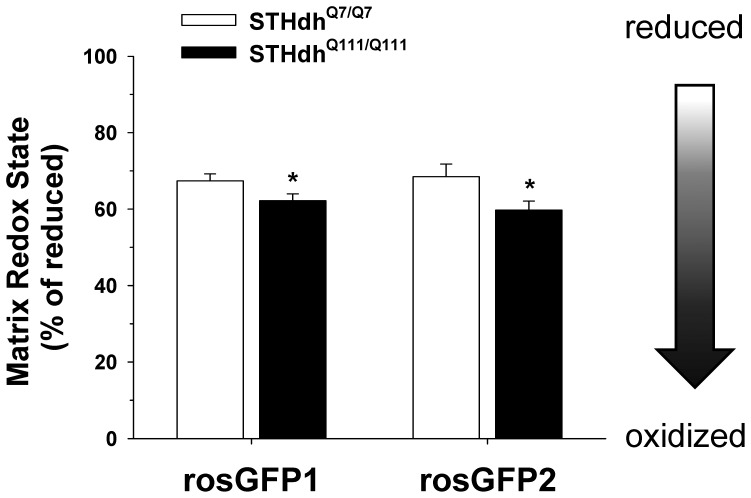
Mitochondria of STHdh^Q111/Q111^ cells are significantly more oxidized. Mitochondrial targeted ROS GFP sensors, rosGFP1 and rosGFP2, were transfected and the relative redox state of mitochondria was assessed as described in materials and methods. The results from both rosGFP1 and rosGFP2 sensors indicate that mitochondrial matrix of STHdh^Q111/Q111^ cells exhibits more oxidized state than that of STHdh^Q7/Q7^ cells in the basal condition. The data was acquired by analysis of more than 40 cells of each cell type from three independent experiments. Data shown are mean ± SE. *P<0.05 vs. STHdh^Q7/Q7^.

Mitochondria play important roles in regulation of cellular redox state and defense against oxidative stress. In addition, our results show that oxidative stress dramatically affects mitochondrial morphologies. Given these data, we next determined if the two striatal cell displayed differences in in the redox states of mitochondria in the basal condition, which could ultimately contribute to different distributions in mitochondrial morphologies. To this end, mitochondrial targeted GFP redox sensors, rosGFP1 or rosGFP2 were transfected into striatal cells [43]. The fluorescence emission at 525 nm was monitored at excitation wavelengths of 470 nm and 400 nm and the ratio of emission intensities (470/400) was used to evaluate mitochondrial matrix redox state as described in methods and materials. The results from these two redox sensors indicate that mitochondria in STHdh^Q111/Q111^ cells are in a more oxidized state compared to STHdh^Q7/Q7^ cells (Fig. 4).

### Nrf2 Signaling Pathway is Impaired in STHdh^Q111/Q111^ Cells

**Figure 5:**
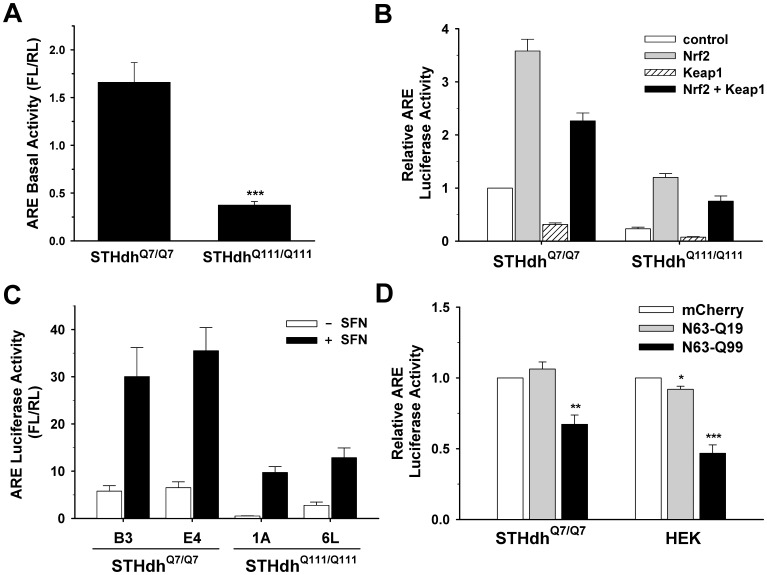
mHtt expression compromises the Nrf2 signaling pathway. *A,* Nrf2 activity was measured in striatal cells using an ARE reporter. The basal activity of Nrf2 is significantly reduced in STHdh^Q111/Q111^ cells compared to STHdh^Q7/Q7^ cells. n = 4 Data shown are mean ± SE. ***P<0.001 vs. STHdh^Q7/Q7^. *B,* An ARE luciferase reporter and a normalizing plasmid phRL-TK were cotransfected with Nrf2, Keap1, or both. Exogenous Nrf2 expression increases the activity of ARE luciferase in STHdh^Q7/Q7^ cells to a much greater extent than in STHdh^Q111/Q111^ cells. The ectopic expression of Keap1 results in a reduction in the ARE activity at the basal level as well as in the presence of exogenous Nrf2 expression in both cell types. n = 4 *C,* ARE activity was examined in the two additional lines for each striatal cell type. STHdh^Q111/Q111^ cell lines (1A and 6L) show significantly reduced ARE activity at the basal level compared to STHdh^Q7/Q7^ cells lines (B3 and E4). Treatment with sulforaphane (SFN), an agonist of Nrf2, increases ARE activity in STHdh^Q7/Q7^ cells to a greater extent than in STHdh^Q111/Q111^ cells. n = 3 *D,* Transient transfection of mHtt with 63 N-terminal amino acids results in a significant reduction in the ARE luciferase activity in STHdh^Q7/Q7^ as well as in HEK cells. n = 3. Data shown are mean ± SE. **P<0.01, ***P<0.001 vs. mCherry.

Nrf2 is a key player in redox regulation and defense mechanisms against ROS [44], [45]. Since STHdh^Q111/Q111^ cells show altered mitochondrial dynamics and increased susceptibility to oxidative stress, we investigated whether the Nrf2 signaling pathway was compromised in STHdh^Q111/Q111^ cells. Nrf2 activity was first assessed using an ARE luciferase reporter. STHdh^Q111/Q111^ cells exhibited significantly reduced ARE activity at the basal level compared to STHdh^Q7/Q7^ cells (Fig. 5*A*). Next, we tested whether the Nrf2 signaling pathway was compromised in STHdh^Q111/Q111^ cells. The overexpression of Nrf2 resulted in a significantly greater increase in ARE activity in STHdh^Q7/Q7^ cells compared to STHdh^Q111/Q111^ cells, indicating an impairment in the Nrf2 signaling pathway in STHdh^Q111/Q111^ cells (Fig. 5*B*). To confirm that the impaired Nrf2 signaling is due to the presence of mHtt, we measured ARE activity in additional striatal cell lines. Two new striatal cell lines expressing mHtt (1A and 6L) exhibited a substantial reduction in ARE activity in the absence or the presence of the Nrf2 agonist sulforaphane compared to two new striatal cell lines expressing Htt (B3 and E4) (Fig. 5*C*). Furthermore, the ectopic expression of N-terminal mHtt led to a significant reduction in ARE activity in STHdh^Q7/Q7^ and HEK cells (Fig. 5*D*). These results demonstrate that the presence of mHtt leads to the disturbance of Nrf2 signaling.

### Nrf2 Activation in Response to Oxidative Stress is Impaired in STHdh^Q111/Q111^ Cells

**Figure 6:**
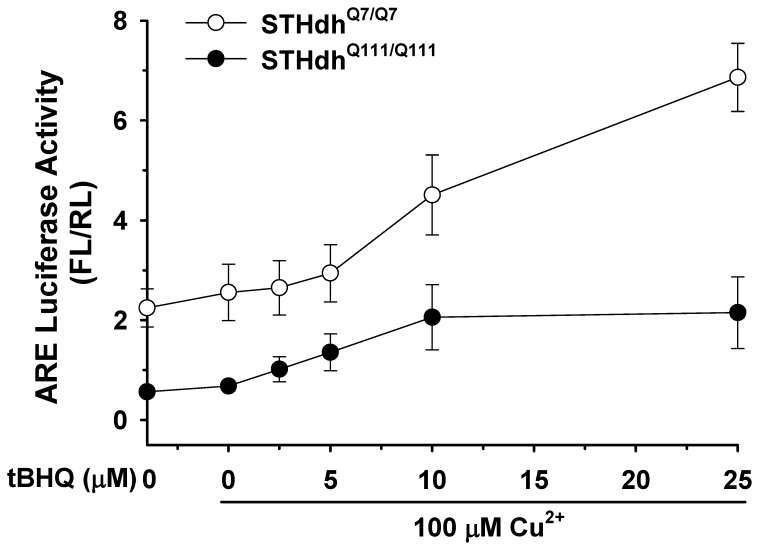
Nrf2 response to oxidative stress is impaired in STHdh^Q111/Q111^ cells. *A*, An ARE reporter and a normalizing plasmid phRL-TK were transfected into striatal cells and oxidative stress was subsequently induced by treatment with tBHQ and Cu^2+^ for 24 h. STHdh^Q7/Q7^ cells show a dramatic increase in ARE activity in response to tBHQ in a dose dependent manner. However, ARE activity of STHdh^Q111/Q111^ cells exhibits only a mild increase at lower concentrations of tBHQ, but no further increase at higher concentrations of tBHQ. n = 3.

Nrf2 is activated by oxidative stress. Therefore we next determined whether mHtt affects Nrf2 activity in response to oxidative stress. Striatal cells were treated with tert-butylhydroquinone (tBHQ) and Cu^2+^ and ARE activity was measured as described in materials and methods. STHdh^Q7/Q7^ cells showed a dramatic increase in ARE activity in a dose dependent manner in response to oxidative stress, while STHdh^Q111/Q111^ cells showed significantly less of an increase in ARE activity at low concentrations of tBHQ and did not exhibit further increases in ARE activity when higher concentrations were used (Fig. 6). This result suggests that the disturbance of Nrf2 signaling renders STHdh^Q111/Q111^ cells more susceptible to oxidative stress.

### Disturbed Signaling of Nrf2 in STHdh^Q111/Q111^ Cells is not due to its Expression Level

**Figure 7:**
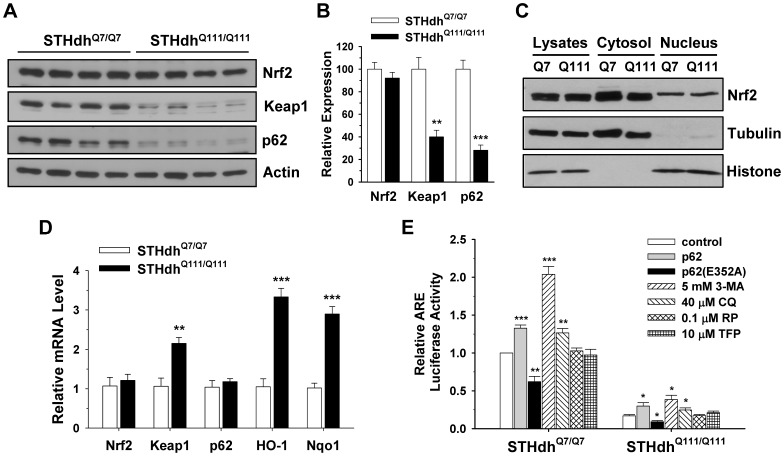
Impaired Nrf2 signaling pathway in STHdh^Q111/Q111^ cells is independent of Nrf2 expression level. *A,* Nrf2 is expressed at a similar level between the two cell types, while Keap1 and p62 are decreased in STHdh^Q111/Q111^ cells. *B,* The quantitative data shows that the expression level of Nrf2 is similar between the two cell types while STHdh^Q111/Q111^ cells exhibit a significant reduction in the expression level of Keap1 and p62. n = 4. Data shown are mean ± SE. **P<0.01, ***P<0.001 vs. STHdh^Q7/Q7^. *C*, Nrf2 levels in the cytosol and nucleus of the STHdh^Q7/Q7^ and STHdh^Q111/Q111^ cells are similar. *D,* The mRNA levels of Nrf2, Keap1, p62, HO-1, and Nqo1 were measured by real-time PCR as described in materials and methods. Nrf2 and p62 levels are similar between the two cell types while STHdh^Q111/Q111^ cells exhibit increased levels of Keap1, HO-1, and Nqo1. n = 4. Data shown are mean ± SE. **P<0.01, ***P<0.001 vs. STHdh^Q7/Q7^. *E,* The relative ARE activity was evaluated in the striatal cells after cotransfection with p62 or an inactive mutant p62(E352A) or in the presence of autophagy effectors. The overexpression of p62 results in a significant increase in ARE activity in both cell types while ARE activity is significantly reduced by the overexpression of p62(E352A) in both cell types. Autophagy inhibition by treatment with 3-MA or CQ results in a significant increase in ARE activity in both cell types with STHdh^Q7/Q7^ cells to a greater extent while autophagy activation by treatment with RP and TFP has no effect. n = 3–4. Data shown are mean ± SE. *P<0.05, **P<0.01, ***P<0.001 vs. control.

Although Nrf2 activity was compromised in the STHdh^Q111/Q111^ cells, there were no detectable differences in protein expression levels between the two cell types (Fig. 7*A*). Since Nrf2 activation is dependent on translocation to the nucleus, we examined Nrf2 levels after subcellular fractionation. There were no obvious differences in Nrf2 levels in the cytosol or nucleus between the two cell types (Fig. 7*C*). In contrast, the basal expression of Keap1 and p62 in STHdh^Q111/Q111^ cells was significantly lower than that of STHdh^Q7/Q7^ cells (Fig. 7, *A* and *B*). Next, we measured mRNA levels by real-time PCR to test whether altered protein levels arise from the change in mRNA levels (Fig. 7*D*). Nrf2 levels were similar between the two cell types, suggesting that altered signaling of Nrf2 in STHdh^Q111/Q111^ cells is not caused by the change of its expression. However, the Nrf2 target genes, HO-1 and Nqo1, were significantly increased in STHdh^Q111/Q111^ cells as compared to STHdh^Q7/Q7^ cells, implying that STHdh^Q111/Q111^ cells are under oxidative stress which may in turn activate defense mechanisms. Upregulation of oxidative stress related genes, including Nrf2 targets, was previously reported in a PC12 cell model in which mHtt exon1 protein was inducibly expressed [46]. However, this PC12 cell model also showed increases in catalase, glutathione peroxidase (GPX1), superoxide dismutase 1 (SOD1), and SOD2, while other studies using different HD models, including our previous study using the striatal cell lines, showed a decrease in these genes [8], 47–49. This discrepancy is likely ascribable to the different forms of mHtt that were expressed (full-length vs highly truncated) as well as the different cell types that were used. Nonetheless, the findings in the different models all indicate that a pathological polyglutamine expansion results in increased oxidative stress. In addition, and unexpectedly, mRNA levels of Keap1 were significantly increased in STHdh^Q111/Q111^ cells, while mRNA levels of p62 were not different between the two cell types (Fig. 7*D*), despite a significant reduction in their protein expression (Fig. 7, *A* and *B*). Since Keap1 and p62 are degraded by autophagy, the reduced protein levels may indicate increased signaling of autophagy in STHdh^Q111/Q111^ cells [50], [51]. p62 is an adaptor molecule that targets polyubiquitylated proteins for degradation by either autophagy or proteasome [52]. p62 can also activate Nrf2 by inhibiting the binding of Keap1 to Nrf2 [53]. In addition, Nrf2 signaling negatively interacts with autophagy as inhibition of autophagy can increase Nrf2 activity [54]. Therefore, we examined whether the modulation of autophagy signaling can differentially influence Nrf2 activity in striatal cells. As previously shown, p62 expression results in a slight but significant increase in Nrf2 activity in both cell types (Fig. 7*E*) [53], [54]. Inhibition of autophagy by treatment with 3-methyladenine (3-MA) or chloroquine (CQ) resulted in a significant increase in Nrf2 activity in both cell types, while the activation of autophagy by treatment with rapamycin (RP) or trifluoperazine (TFP) did not change Nrf2 signaling (Fig. 7*E*). However, the ectopic expression of p62 or inhibition of autophagy failed to recover Nrf2 signaling in STHdh^Q111/Q111^ cells to the levels observed in STHdh^Q7/Q7^ cells. Together, this result suggests that p62 and autophagy signaling are not the major contributing factors to impaired Nrf2 signaling in STHdh^Q111/Q111^ cells.

### Nrf2 Expression Attenuates the Fragmentation of Mitochondria

**Figure 8:**
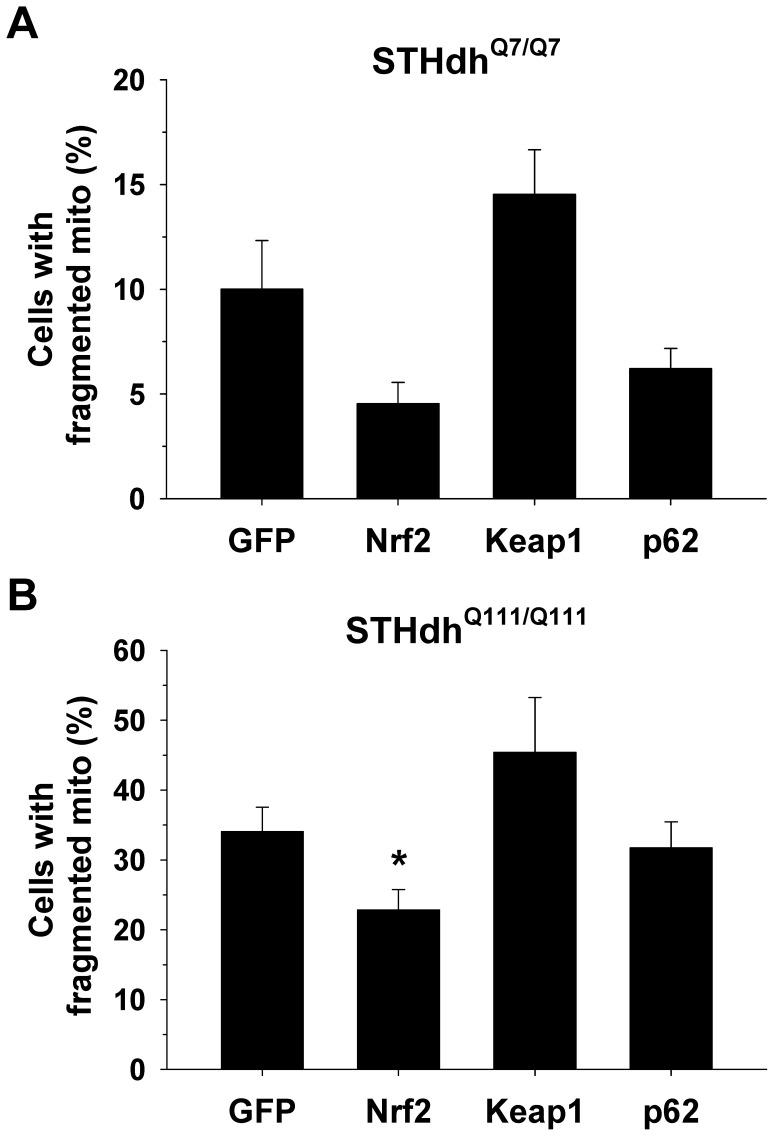
Nrf2 expression attenuates the fragmentation of mitochondira in striatal cells. Striatal cells were transfected with mito-mCherry as well as GFP, Nrf2, Keap1, or p62. Two days after transfection, cells were fixed with 4% paraformaldehyde and the morphology of mitochondria was assessed. *A-B*, The exogenous expression of Nrf2 reduces the number of cells with fragmented mitochondria in the striatal cells. In contrast, Keap1 expression results in a strong trend of increase in the number of cells with fragmented mitochondria in the both cell types, while p62 expression does not seem to have any effect. More than 200 cells of each cell type were assessed for each independent experiment. n = 4. Data shown are mean ± SE. *P<0.05 vs. GFP.

Given that STHdh^Q111/Q111^ cells display an increase in fragmented mitochondria and more oxidized mitochondria, we investigated whether Nrf2 expression modifies mitochondrial morphology (Fig. 8). Striatal cells were transfected with mito-mCherry and Nrf2, Keap1, p62, or GFP as a control. Nrf2 expression results in a significant reduction in fragmented mitochondria in STHdh^Q111/Q111^ cells and a similar trend of reduction in STHdh^Q7/Q7^ cells. Conversely, Keap1 expression appeared to increase the number of cells with fragmented mitochondria, although it was not statistically significant. The expression of p62 did not lead to any obvious alteration in mitochondrial morphology in either cell type. This result suggests that oxidative stress is a contributing factor to mitochondrial morphology which can be in part modulated by Nrf2 signaling. This observation is in line with the previous study showing that oxidative stress caused by 3-NP increases mitochondrial fragmentation, leading to neuronal cell death and that blockade of ROS abolishes mitochondrial fragmentation as well as cell death [55].

### Autophagy Signaling is Highly Activated in STHdh^Q111/Q111^ Cells

**Figure 9:**
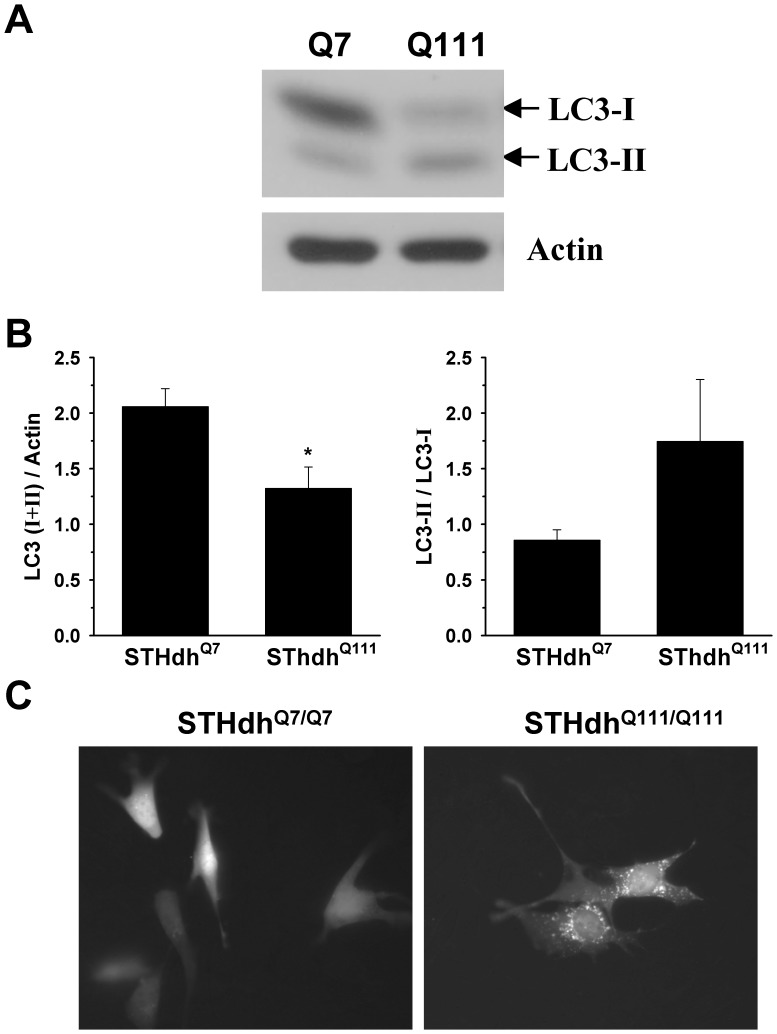
STHdh^Q111/Q111^ cells exhibit an increase in autophagy activity. *A–B,* The increase of LC3-I into LC3-II is a typical indicator of the autophagy. The total amount of LC3 including type I and II is significantly higher in STHdh^Q7/Q7^ cells than STHdh^Q111/Q111^ cells. In addition, the ratio of type II to type I seems to be greater in STHdh^Q111/Q111^ cells than in STHdh^Q7/Q7^ cells. n = 4. Data shown are mean ± SE. *P<0.05 vs. STHdh^Q7/Q7^. *C,* Cells were transfected with GFP-LC3 (microtubule-associated protein 1 light chain 3) and 24 h later GFP fluorescence images were monitored. LC3-I is a cytosolic form of LC3 and LC3-II is a membrane-bound form of LC3 that is implicated in autophagy. The localization of GFP-LC3 is the diffused cytosolic pattern in most of STHdh^Q7/Q7^ cells, while STHdh^Q111/Q111^ cells exhibit punctuate pattern of GFP-LC3 that indicates the active autophagic vesicles.

We next examined whether autophagy regulation is altered in STHdh^Q111/Q111^ cells compared to STHdh^Q7/Q7^ cells. First, we looked at the conversion of LC3-I into LC3-II as an indicator of autophagy activation. Although the total level of LC3 is significantly reduced in STHdh^Q111/Q111^ cells, the ratio of LC3-II to LC3-I appears to be increased in STHdh^Q111/Q111^ cells compared to STHdh^Q7/Q7^ cells (Fig. 9, *A* and *B*). To further assess the autophagy activation, we transfected GFP-LC3 into striatal cells and monitored the cellular distribution of GFP-LC3. As the activation of autophagy increases, GFP-LC3 relocates from the cytosol into autophagosome, resulting in the punctate pattern. We found that STHdh^Q111/Q111^ cells show numerous GFP-LC3 positive punctae, while STHdh^Q7/Q7^ cells showed only a few GFP-LC3 positive punctae (Fig. 9*C*). Together, these results indicate that STHdh^Q111/Q111^ cells exhibit highly activated autophagy signaling.

## Discussion

Mitochondrial impairment is emerging as a contributing factor to the pathogenesis of HD. A growing number of studies using mouse and cell models for HD, as well as tissues from HD patients, have shown that mHtt causes mitochondrial functional impairment such as hypersensitivity to Ca^2+^ induced permeability transition pore opening, reduced Ca^2+^ buffering capacity, deficits in the electron transport chain complexes and impaired mitochondrial bioenergetics [2]–[4]. An important factor mediating mitochondrial function is mitochondrial dynamics. An appropriate balance in mitochondrial fusion and fission is essential for cells to maintain metabolic states and homeostasis, and to respond to cellular stresses [12]. Recently, it demonstrated that the presence of mHtt leads to mitochondrial fragmentation in *in vitro* and *in vivo* HD models including HeLa cells and rat cortical neurons expressing mHtt, fibroblasts and lymphoblasts from HD patients, striatal progenitor cell lines, and YAC128 mouse [14], [19], [56]. Our finding of an increase in fragmented mitochondria with decreased level of tubular mitochondria in STHdh^Q111/Q111^ cells, as well as increased fragmented mitochondria in the striata of R6/2 mice, is in line with these previous studies. In addition, studies from two recent papers indicate that enhanced activation of Drp1 by mHtt causes mitochondrial fragmentation. Song et al. suggested that mHtt directly interacts with Drp1, which in turn increases its activity, whereas Costa et al. proposed the increased activity of calcineurin, a phosphatase which dephosphorylates Drp1, as a cause of increased Drp1 activity [14], [19]. Increased steady state levels of mitochondrial fragmentation can be induced by increased fission, reduced fusion, or both. Their studies strongly support the idea of increased fission as one possible route to mitochondrial fragmentation. However, these studies do not rule out reduced fusion as an alternative pathway that contributes to mitochondrial fragmentation. To our knowledge we provide the first evidence that the expression of mHtt in striatal precursor cells negatively impacts mitochondrial fusion. Furthermore, we found that the overall levels of Opa1 are significantly reduced in STHdh^Q111/Q111^ cells compared to STHdh^Q7/Q7^ cells, suggesting that reduced Opa1 could be causally involved in the reduced fusion process. Moreover, it has been shown that Opa1 expression attenuates the apoptosis in HD models, but Mfn1 expression did not despite correction of mitochondrial fragmentation, suggesting deficits in Opa1 may be a contributing factor to the mitochondrial fragmentation in HD [19].

It has been speculated that enhanced oxidative stress may play a significant role in HD pathogenesis [5], [57]. Mitochondria play important roles in the defense against oxidative stress. A previous study showed that oxidative stress increases mitochondrial fragmentation in HeLa cells expressing mHtt [56]. In contrast, we observed a rapid increase in the population of STHdh^Q111/Q111^ cells containing swollen mitochondria rather than fragmented mitochondria in response to oxidative stress. STHdh^Q7/Q7^ cells actually display an increase in fragmented mitochondria in response to oxidative stress, followed by a slow increase in swollen mitochondria. This result implies that upon cellular stresses, mitochondrial fragmentation would be a prerequisite for mitochondria swelling, an event that leads to the pathogenesis. Dysregulation of transcriptional processes that govern mitochondrial biogenesis and antioxidant system would result in mitochondrial impairment that would be exacerbated by cellular oxidative stress. PGC-1α is a transcriptional coactivator that plays important roles in mitochondrial biogenesis as well as defense mechanism against oxidative stress and was discovered to be repressed in models of HD [58]. In addition, we previously found that STHdh^Q111/Q111^ cells exhibit a deficit in the PPARγ pathway [8], [11] that regulates the expression of genes involved in mitochondrial function, ROS regulation, and fatty acid metabolism [59], [60]. Given that transcriptional dysregulation is tightly linked with mitochondrial impairment as well as defects in antioxidant response, we sought to find additional transcriptional regulator that play important roles in these pathways and may be compromised in HD. Nrf2 has been considered as a key regulator in ROS defense mechanism and mitochondrial function. We discovered that Nrf2 signaling is severely compromised by the presence of mHtt. More importantly, STHdh^Q111/Q111^ cells fail to activate Nrf2 in response to oxidative stress. This result provides important insight into a potential contributing factor to the apparent impaired ability of cells to cope with oxidative stress when mHtt is present.

Two well known regulators of Nrf2 signaling are Keap1 and p62. Keap1 is an inhibitor that binds to Nrf2, leading to degradation through the ubiquitin-proteasome system. p62 is an activator that competes with Nrf2 for Keap1 binding and sequesters Keap1 into aggregates [53], [54]. To gain insights into the mechanism of how Nrf2 signaling is compromised in STHdh^Q111/Q111^ cells, we examined the protein levels of Nrf2, Keap1, and p62 in the two striatal cell lines. We did not detect any difference in Nrf2 levels between the two cell types. Of interest, Keap1 and p62 were significantly downregulated in STHdh^Q111/Q111^ cells. This result indicates that the reduced activity of Nrf2 is not due to decreased levels of Nrf2 or increased levels of Keap1. p62 has been reported to be regulated by binding of Nrf2 to an ARE in the p62 promoter [61], but this is unlikely to be responsible for the reduction in p62 protein in the STHdh^Q111/Q111^ cells as mRNA levels for p62 were the same for both cell lines. It is noteworthy that Keap1 and p62 are selective substrates for autophagy [50], [53]. It is plausible therefore, that reduced levels of Keap1 and p62 in STHdh^Q111/Q111^ cells may stem from enhanced activation of autophagy. We tested if STHdh^Q111/Q111^ cells show an increase in autophagy activity. As expected, autophagy signaling is highly activated in STHdh^Q111/Q111^ cells. Consistent with this finding, inhibition of autophagy by treatment with 3-MA or CQ resulted in a significant increase in Nrf2 activity in both cell types. Autophagy inhibition may lead to increased level of p62 which in turn inhibits Keap1 binding to Nrf2. Nonetheless, Nrf2 activity in STHdh^Q111/Q111^ cells was not increased by inhibition of autophagy to the same extent as in STHdh^Q7/Q7^ cells. This result suggests that hyperactivation of autophagy signaling in STHdh^Q111/Q111^ cells is likely a partial, but not a major, contributing factor to compromised Nrf2 activity.

The remaining question is what could be other contributing factors to disrupted Nrf2 signaling in STHdh^Q111/Q111^ cells? We sought to understand the underlying mechanism by which mHtt results in impairment in Nrf2 signaling. GSK3β has been reported to inactivate Nrf2 signaling by direct phosphorylation of Nrf2, a target of an E3 ubiquitin ligase adaptor β-TrCP that leads to proteasomal degradation, or by phosphorylation of Fyn, a kinase that leads to nuclear export and degradation of Nrf2 by phosphorylation of Nrf2 [62]–[64]. These studies suggest that GSK3β activation could be a causal factor reducing Nrf2 signaling. However, a previous study showed enhanced Akt activation which results in specific inactivation of GSK3β in STHdh^Q111/Q111^ cells and striatum of Hdh^Q111/Q111^ knockin mouse [65]. This led us to rule out GSK3β as a possible player that negatively influences Nrf2 signaling in STHdh^Q111/Q111^ cells. Previous studies demonstrated that CBP/p300 directly binds Nrf2, resulting in acetylation of Nrf2, a state that enhances Nrf2 transcriptional activity [66], [67]. CBP/p300 has been shown to interact with mHtt, which results in inhibition of their acetyltransferase activity [68], [69]. Therefore, it is conceivable that interference of CBP/p300 by mHtt could be a crucial factor that leads to disrupted Nrf2 signaling in STHdh^Q111/Q111^ cells. Investigation of this hypothesis in the future may be fundamental to understanding the exact molecular mechanism by which Nrf2 signaling is compromised in STHdh^Q111/Q111^ cells and to provide insights into development of therapeutic strategies of HD.

In summary, for the first time we provide evidence that mitochondrial fusion is impaired in an HD striatal precursor cell model. As a result, STHdh^Q111/Q111^ cells display an increased population of cells with fragmented mitochondria compared to STHdh^Q7/Q7^ cells. In addition, STHdh^Q111/Q111^ cells are more susceptible to oxidative stress, leading to increased levels of swollen mitochondria. We also identified disrupted Nrf2 signaling in STHdh^Q111/Q111^ cells which is likely to contribute increased mitochondrial fragmentation and functional impairment and thus increased susceptibility of STHdh^Q111/Q111^ cells to oxidative stress. This study provides important insights into involvement of defects in mitochondrial dynamics in the pathogenic mechanisms of mHtt.

## Materials and Methods

### Ethics Statement

All animal protocols have been approved by the UCAR at the University of Rochester (UCAR# 2006-102R).

### Materials and Antibodies

Sulforaphane (SFN), 3-methyladenosine (3-MA), and rapamycin (RP) were purchased from Calbiochem. tert-butylhydroquinone (tBHQ), chloroquine (CQ), and trifluoperazine (TFP) were obtained from Sigma. All other chemicals were purchased from Sigma (St. Louis, MO, USA), if not otherwise indicated. Anti-Nrf2, anti-Keap1, anti-Mfn2, anti-p62/SQSTM1, anti-GFP, and anti-actin antibodies were purchased from Abcam, Proteintech, Sigma, Biomol, Roche, and Chemicon, respectively. Antibodies for pDrp1 (S616) and LC3 were acquired from Cell Signaling Technology. Antibodies for Opa1 and Drp1 were purchased from BD Biosciences.

### Plasmid Constructs

rosGFP1 and rosGFP2 were kindly provided from Dr. Roderick A. Capaldi [43]. pEGFP-mitoGFP was a kind gift from Dr. Yisang Yoon. pGL3-ARE was a kind gift from Dr. Martin Leonard [70]. pcDNA3-EGFP-C4-Nrf2 and pcDNA3-HA2-Keap1 were obtained from Addgene [71]. Myc-p62 and myc-p62 (G352A) were kindly provided by Dr. Terje Johansen [61]. Mito-mCherry and photoconvertible mitoDendra were constructed by subcloning the mCherry from pFH6.II-mCherry (a kind gift of Dr. Keith Nehrke) and the Dendra from pDendra2-C (Evrogen) using PCR. The resulting PCR products were ligated into the AgeI and BsrG sites of pEGFP-mitoGFP. GFP-LC3 was previously described [72]. Expression constructs of truncated huntingtin with N-terminal 63 amino acids, pcDNA3.1-N63-Q19 and pcDNA3.1-N63-Q99 have been previously reported [73].

### Cell Culture

The immortalized homozygote striatal cell lines, STHdh^Q7/Q7^ (the original one, B3, E4) and STHdh^Q111/Q111^ (the original one, 1A, 6L), made from striatal primordia of E14 mouse embryos expressing Htt with 7 polyQ or mHtt with 111 polyQ were kindly provided by Dr. Marcy MacDonald [74]. Cells were cultured in DMEM containing 25 mM glucose and 4 mM glutamine (Invitrogen) supplemented with 4% fetal bovine serum (FBS, HyClone, Waltham, MA, USA) and 4% bovine growth serum (BGS, HyClone), and 100 units/ml penicillin and 100 µg/ml streptomycin (Invitrogen) and maintained at 33°C and 5% CO_2_. HEK 293-TN cells were grown in DMEM supplemented with 10% FBS, and 100 units/ml penicillin and 100 µg/ml streptomycin. Cells were maintained at 37°C in a humidified atmosphere containing 5% CO_2_.

### Cell Fusion Assay

To monitor mitochondrial fusion, cell fusion was carried out as previously described with modification [7], [75]. In brief, STHdh^Q7/Q7^ or STHdh^Q111/Q111^ cells were plated in two of 35 mm dish. The next day, mitoGFP or mito-mCherry was transfected in each dish using Lipofectamine 2000 following the manufacturer’s protocol. After 24 h, cells in both dishes were trypsinized, combined, and plated together onto 2–3 wells of 12 well plate containing a glass-coverslip in each well. The next day, cells were treated with 50 µg/ml of cycloheximide for 30 min prior to cell fusion to prevent protein synthesis. To fuse cells, prewarmed 50% w/v polyethylene glycol (PEG) 4000 in G-HBSS (20 Hepes, pH 7.4, 137 NaCl, 5 KCl, 0.5 KH_2_PO_4_, 0.5 Na_2_HPO_4_, 10 NaHCO_3_, 0.01 glycine; in mM), 2 mM CaCl_2_, 0.6 mM MgCl_2_, and 10 mM glucose was added to cells for 90 s. The cells were then washed gently four times with G-HBSS, placed back in culture medium containing cycloheximide, and returned to the incubator at 33°C for 3 h. Cells were fixed with fresh 4% paraformaldehyde containing 0.25% glutaraldehyde for 20 min at room temperature. The percentage of mitochondrial fusion was calculated by colocalization of mitoCherry and mitoGFP using image J software.

### Mitochondrial Fusion Assay with MitoDendra

Striatal cells were plated on 12 well plates containing a glass coverslip in each well. MitoDendra was transfected 1–2 d after plating. The next day, a glass coverslip was placed on the microscope (Observer D1, Zeiss) and the green/red fluorescence of mitochondria was observed with a 63× oil objective. To photo-convert a small area in mitochondria was chosen and the aperture was minimized. The green fluorescence was photoconverted into the red fluorescence by illuminating mitochondria using a 400 nm LED with 100% power controlled by Colibri system (Zeiss) for 6 min. The mitochondrial images were taken by a digital CCD camera (ORCA-ER, Hamamatsu Photonics) every 30 s for 30 min with 2 digital gain factor. The green fluorescence was obtained by using a 470 nm LED with 3–5% power for 20–30 ms exposure and a 525/20 nm emission filter. The red fluorescence was imaged with a 530 nm LED with 100% power for 10–20 ms exposure and a 630/75 emission filter. The time-lapse merged images from green and red channels were generated using image J. A green mitochondrion which encounters a red mitochondrion resulting in colocalization (yellow mitochondrion) was considered as a mitochondrial fusion event.

### Ratiometric Measurement of Mitochondrial Matrix Redox State using Mitochondrial Targeted GFP Sensors

Cells were plated on glass coverslips and transfected with rosGFP1, or rosGFP2 [43] using Lipofectamine 2000 according to manufacturer’s instruction. Th next day, media were removed and replaced with G-HBSS for 30 min at 37°C. The fluorescence from mitochondria was imaged at 63× magnification on an Observer D1 microscope (Zeiss) coupled with a digital CCD camera (ORCA-ER, Hamamatsu). The emission was detected through a 525/20 nm filter with excitation consecutively from a 470 nm LED and a 400 nm LED controlled by a Colibri system (Zeiss) with the same exposure time. Fluorescence images were background corrected, mitochondrial images were selected, and emission intensities from 470 nm and 400 nm were acquired using Image J software. The ratio of emission intensities (470/400) was calculated in Microsoft Excel software. To generate a standard curve for the measurement of matrix redox state, cells were treated for 30 min in G-HBSS containing 5 mM H_2_O_2_ or 10 mM DTT to obtain the maxima of oxidation and reduction, respectively. The standard curves were generated using Sigmaplot (Systat Software).

### Western Blot Analysis

Cells were washed with ice-cold phosphate-buffered saline (PBS) and lysed with modified RIPA buffer (50 mM Tris-HCl pH 7.4, 150 mM NaCl, 1% Triton X-100, 0.4% SDS, 0.2% sodium deoxycholate, 5% glycerol, 1 mM EDTA, 20 mM NaF, 2 mM Na_3_VO_4_) containing protease inhibitors (1 mM PMSF, 10 µg/ml leupeptin, 10 µg/ml aprotinin, 10 µg/ml pepstatin). The lysates were sonicated, cleared by centrifugation, and assayed to determine protein concentration using BCA assay (Pierce Biotechnology, Waltham, MA, USA). Proteins (10–100 µg) were separated by SDS-PAGE and transferred to nitrocellulose membrane. The membrane was blocked with 5% skim milk in Tris-buffered saline containing 0.05% Tween 20 (TBST), and incubated with the specific antibodies in TBST containing 2% BSA or skim milk at 4°C overnight. After washing three times, HRP-conjugated secondary antibody (1∶3000) in TBST containing 5% skim milk was applied and the blot was visualized by chemiluminescence. The intensity of immunoreactive bands was quantified by using Image J software.

### Nuclear Fractionation

Nuclear fractionation was carried out as previously described [76] with slight modifications. Briefly, STHdh^Q7/Q7^ and STHdh^Q111/Q111^ cells were washed twice, harvested in ice cold PBS and the cell pellets were resuspended in lysis buffer (10 mM Tris, pH 7.5, 10 mM NaCl, 3 mM MgCl_2_, 0.05% Nonidet P-40, 1 mM EGTA) with protease inhibitors (0.1 mM phenylmethylsulfonyl fluoride, and 10 µg/ml of each of aprotinin, leupeptin, pepstatin A). A sample was taken and used as whole cell lysate. The cells were triturated followed by centrifugation at 380 × g for 5 min at 4°C. The supernatants were collected and used as the cytosolic fractions. The pellets were washed once in lysis buffer and twice in wash buffer (30 mM sucrose, 10 mM Pipes, pH 6.8, 3 mM MgCl_2_, 1 mM EGTA, 25 mM NaCl) with protease inhibitors. The crude nuclei were overlaid on the top of 0.8 M sucrose with protease inhibitors, and spun at 1200 × g for 10 min at 4°C. The pellets were collected and resuspended in buffer B (300 mM sucrose, 10 mM Pipes, pH 6.8, 3 mM MgCl_2_, 1 mM EGTA, 25 mM NaCl, 0.5% Triton X-100) with protease inhibitors and used as the nuclear fractions. 20 µg of protein from each sample was visualized by immunoblot analysis.

### Dual-luciferase Reporter Assays

Cells were plated in 24 well plates. The next day, a reporter plasmid pGL3-ARE and a normalizing plasmid phRL-TK (Promega) were transfected using Lipofectamine 2000. The next day, cells were treated with vehicle control or drug. After 24 h, cells were lysed with Passive lysis buffer (Promega), and the reporter activity was measured using the Dual-Luciferase Reporter Assay System (Promega). The reporter activity from Firefly luciferase was normalized with the Renilla luciferase activity.

### RNA Isolation, Reverse Transcription, and Real-time PCR

Cells were plated in 60 mm dish and total RNA was extracted using TRIzol (Invitrogen) according to the manufacturer’s instruction. Extracted total RNA was treated with RNase free- DNaseI Amplification Grade (Invitrogen) to remove contaminating DNA. Two micrograms of total RNA was reverse transcribed using SuperScriptIII reverse transcriptase and random hexamers (Invitrogen). The reaction mixture was diluted with 500 µl of DEPC-treated H_2_O. The PCR reaction was prepared in triplicate containing 10 µl of diluted cDNA, 2.5 µl of 2.5 µM primer mixture (forward and reverse), and 12.5 µl of SYBR GreenER qPCR SuperMix (Invitrogen) in 96 well optical PCR reaction plate (Bio-Rad). PCR reactions were performed in MyiQ real-time PCR system (BioRad). Amplification conditions consisted of an initial hot start at 95°C for 10 min followed by amplification of 45 cycles (95°C for 15 s, 60°C for 20 s, and 72°C for 40 s). Melting curve analysis was performed immediately after amplification. The relative amount of mRNAs was calculated by using the ΔΔCt (Ct, threshold cycle) method. The Ct value of β-actin was used for normalization. The sequences of primers are as follows. Nrf2-F: TCACACGAGATGAGCTTAGGGCAA, Nrf2-R: TACAGTTCTGGGCGGCGACTTTAT; Keap1-F: TTAAAGCCATGTTCACCAACGGGC, Keap1-R: TTAAAGCCATGTTCACCAACGGGC; p62-F: TGAAACATGGACACTTTGGCTGGC, p62-R: ACATTGGGATCTTCTGGTGGAGCA; HO-1-F: TCGTGCTCGAATGAACACTCTGGA, HO-1-R: TGTGTTCCTCTGTCAGCATCACCT; Nqo1-F: ATTGTGGCCGAACACAAGAAGCTG, Nqo1-R: TAGGCAAATCCTGCTACGAGCACT; β-actin-F: TGTGATGGTGGGAATGGGTCAGAA, β-actin-R: TGTGGTGCCAGATCTTCTCCATGT.

### Measurement of Mitochondria in R6/2 and Wild Type Mouse Brain

Approximately 13 weeks old transgenic R6/2 mice overexpressing exon 1 of mHtt and their wild type littermates were transcardially perfused with paraformaldehyde/glutaraldehyde. Their brains were subsequently processed and sectioned at 100 µm thickness. Striatal sections were incubated with an antibody (EM48, Chemicon) recognizing huntingtin followed by a secondary antibody conjugated to 1.4 nm gold particles. Sections were silver intensified, osmicated in 1% OsO4, dehydrated and embedded in Eponate 12. Ultrathin sections (50 nM) were cut and counterstained with 5% aqueous uranyl acetate followed by Reynolds lead citrate prior to being examined and captured by the Transmission Electron Microscope Hitachi 7650. Each mitochondrion was measured using Image-Pro. All mitochondria (20–30) in a given cell and approximately 20 cells/animal from several striatal sections were analyzed.

### Statistical Analysis

Data were expressed as mean ± SE (standard error) and analyzed using Student's *t* test. Statistical significance was considered when a *P* value was <0.05.

## Supporting Information

Figure S1 **Quantitative measurement of mitochondrial size and shape.** Electron micrographs of striatal neurons from non-transgenic (A) and transgenic (Tg) R62 mice (B) were captured using electron microscopy. Nuclear aggregates in Tg animals were identified by an antibody against huntingtin protein (arrow). Each individual mitochondrion (red arrowhead) was measured using Image-Pro. All mitochondria (20–30) in a given cell and ∼20 cells/animal from more than one striatal sections were analyzed. Data represent % of total mitochondria (∼ 600 from 2 mice/genotype) ±SEM. Quantitative measurements of mitochondria size were grouped into different size bins (C) and or expressed as aspect ratio (D) (major/minor axes, an index of roundness with values approach 1 as the structure becomes more circular) and analyzed using t-test.
(TIF)
