# Supplementary figures and images for: Impaired Mitochondrial Dynamics and Nrf2 Signaling Contribute to Compromised Responses to Oxidative Stress in Striatal Cells Expressing Full-Length Mutant Huntingtin
Source: PLoS One. 2013 Mar 1;8(3):e57932. doi: 10.1371/journal.pone.0057932 (PMC3585875; doi:10.1371/journal.pone.0057932)

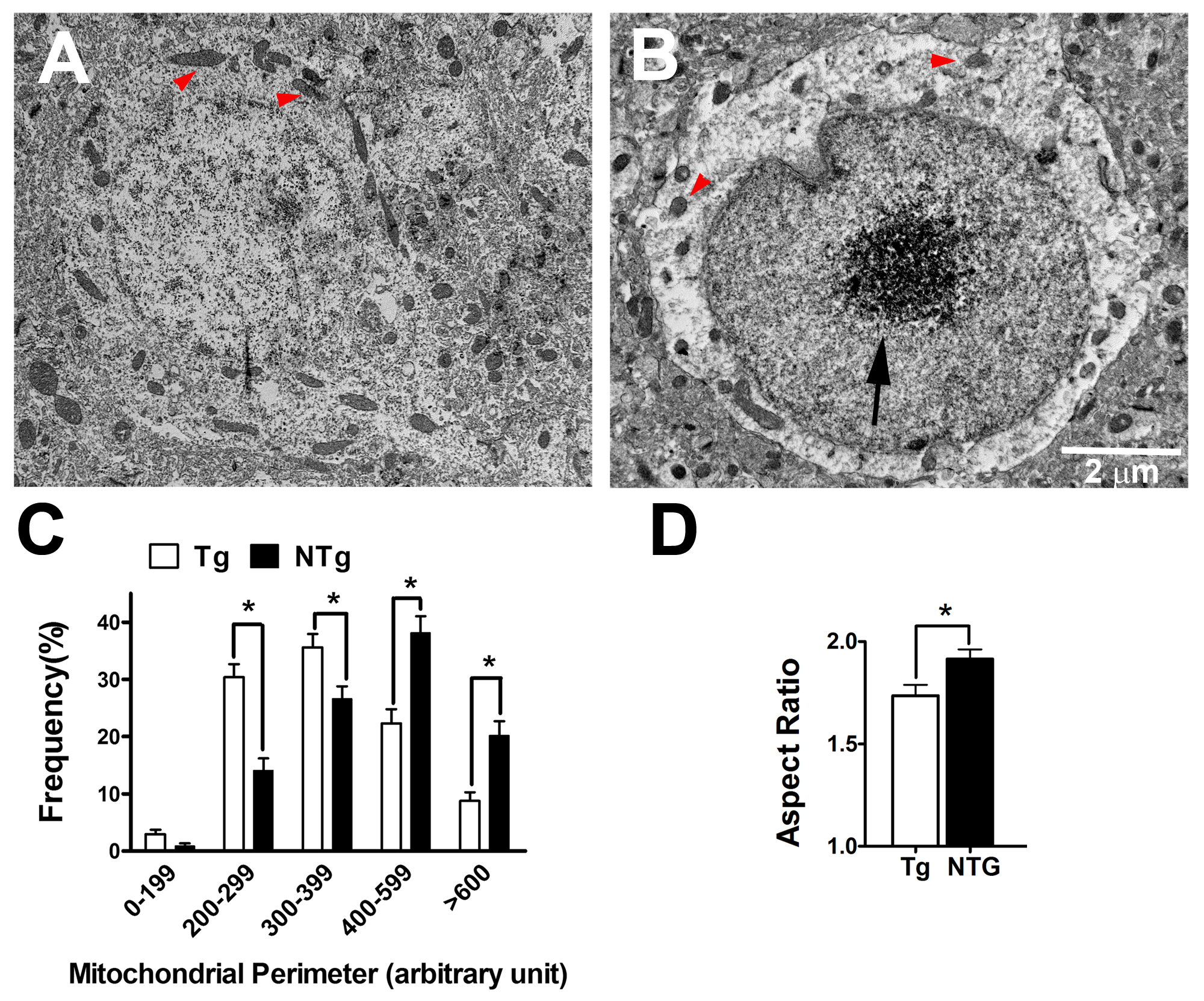

Supplement: Figure S1 — Quantitative measurement of mitochondrial size and shape. Electron micrographs of striatal neurons from non-transgenic (A) and transgenic (Tg) R62 mice (B) were captured using electron microscopy. Nuclear aggregates in Tg animals were identified by an antibody against huntingtin protein (arrow). Each individual mitochondrion (red arrowhead) was measured using Image-Pro. All mitochondria (20–30) in a given cell and ∼20 cells/animal from more than one striatal sections were analyzed. Data represent % of total mitochondria (∼ 600 from 2 mice/genotype) ±SEM. Quantitative measurements of mitochondria size were grouped into different size bins (C) and or expressed as aspect ratio (D) (major/minor axes, an index of roundness with values approach 1 as the structure becomes more circular) and analyzed using t-test. (TIF) [file pone.0057932.s001.tif]
